# Tongue swab Xpert MTB/RIF Ultra for tuberculosis diagnosis in Ethiopian prisons

**DOI:** 10.1128/spectrum.00840-26

**Published:** 2026-08-17

**Authors:** Kelemework Adane, Russell R. Kempker, Kidist Bobosha, Abyot Meaza, Henry M. Blumberg

**Affiliations:** 1 Department of Microbiology, Immunology and Parasitology, College of Health Sciences, Addis Ababa University 37602, Addis Ababa, Ethiopia; 2 Department of Medicine, Division of Infectious Diseases, Emory University School of Medicine 12239, Atlanta, Georgia, USA; 3 Armauer Hansen Research Institute, Mycobacterial Diseases Research Directorate, Addis Ababa, Ethiopia; 4 Ethiopian Public Health Institute 128164, Addis Ababa, Ethiopia; 5 Department of Global Health, Emory University Rollins School of Public Health 25798, Atlanta, Georgia, USA; 6 Department of Epidemiology, Emory University Rollins School of Public Health 25798, Atlanta, Georgia, USA; Maryland Department of Health, Baltimore, Maryland, USA; University of Utah Health, Salt Lake City, Utah, USA; The Hospital for Sick Children, Toronto, Canada

**Keywords:** diagnostic accuracy, incarcerated individuals, tongue swab testing, tuberculosis, Ultra

## Abstract

Tuberculosis (TB) diagnosis remains a major challenge in Ethiopian prisons, where access to diagnostics is limited. Current TB diagnostic strategies in Ethiopian prisons rely primarily on sputum-based testing. However, sputum collection is often challenging due to logistical barriers and infection-control concerns. Tongue swabs offer a promising non-sputum alternative. We assessed the diagnostic accuracy and feasibility of tongue swab testing using Xpert MTB/RIF Ultra (Ultra) for diagnosing pulmonary TB in Ethiopian prisons. Incarcerated men (≥18 years) presenting with a cough of any duration were enrolled. Two tongue swabs were collected from each participant: an early-morning swab and a second swab collected 30 min later. Tongue swabs were processed using a 2:1 sample reagent (SR) to sample ratio and tested with Ultra. Diagnostic accuracy was assessed against a composite microbiological reference standard (MRS) comprising sputum Ultra and liquid culture. Of 221 participants included in the final analysis, 34 (15.4%) had TB. Compared with the MRS, tongue swab Ultra detected 21 cases, yielding a sensitivity of 61.8% (95% CI: 44.7%–76.6%) and a specificity of 100.0%. Sensitivity compared with sputum Ultra was 63.6% (95% CI: 45.1%–79.6%), with 100.0% specificity. Sensitivity varied according to sputum Ultra bacillary load: 100.0% (9/9) for medium or high loads, 70.6% (12/17) for low or very low loads, and 0.0% (0/7) for “trace” loads. Tongue swab testing with Ultra was feasible and showed modest sensitivity but excellent specificity for TB diagnosis in Ethiopian prisons, supporting its use as a complementary diagnostic approach to improve case detection.

**IMPORTANCE:**

This study is among the few to evaluate tongue swab testing using Ultra for TB diagnosis in a sub-Saharan African prison setting. It demonstrates the diagnostic performance and operational feasibility of this approach in a high-burden prison context. It supports the potential integration of non-sputum-based diagnostic approaches into prison TB diagnostic systems. Tongue swab testing using Ultra could improve access to TB diagnostics within existing case-finding algorithms, particularly by identifying individuals who are unable or unwilling to produce sputum.

## OBSERVATION

Tuberculosis (TB) is the leading infectious cause of death from a single pathogen (1). Incarcerated populations are disproportionately affected, with TB prevalence in prisons estimated at 3.5% globally—up to 30 times higher than in the general population (2–4). In Ethiopia, the burden is particularly high, with a pooled TB prevalence of 9.8% among incarcerated individuals (5). Current TB diagnostic strategies in prisons in Ethiopia rely primarily on sputum-based testing. However, sputum collection is often challenging because of logistical barriers, limited privacy, associated stigma, and the presence of human immunodeficiency virus (HIV) co-infection, which is associated with paucibacillary disease and reduced sputum production (6–8). Reliance on sputum-based testing may lead to missed TB cases and delayed diagnosis.

These challenges highlight the need for alternative approaches that can increase access to TB diagnosis in prisons. The World Health Organization recommends systematic TB screening in prisons and has prioritized the development of non-sputum-based diagnostic strategies (9). Tongue swabs have emerged as a promising non-sputum specimen for TB diagnosis because they are easy to collect and require minimal infection control measures (10). Tongue swab testing using Xpert MTB/RIF Ultra (Ultra; Cepheid, Sunnyvale, CA, USA) has shown sensitivities ranging from 45.0% to 78.1% in symptomatic adults (11–16). However, this approach has not been systematically evaluated for TB diagnosis in prison populations. We evaluated the diagnostic accuracy and feasibility of tongue swab testing using Ultra for the diagnosis of pulmonary TB among incarcerated men in Ethiopian prisons.

We conducted a cross-sectional study at Kality Federal Prison and Kilinto Detention Center in Addis Ababa and at Ziway Prison (~170 km south of Addis Ababa). Eligible participants were adult men (≥18 years) with a cough of any duration, with or without other TB-related symptoms, who provided informed consent and were able to provide sputum and tongue swab samples. Individuals who had received anti-TB treatment for more than two days before enrollment were excluded. Participants were recruited through active symptom screening, contact tracing, and self-initiated visits to prison TB clinics. Screening and enrollment were conducted by two study nurses between January 15 and October 31, 2025.

Two tongue swabs were collected from each participant: the first early in the morning and the second 30 min later, before eating, drinking, and performing oral hygiene procedures. Each tongue swab was obtained by brushing the mid-dorsum of the tongue 10 times using FLOQSwabs (Tianzhu Sun Time Biotechnology, China). On each study day, tongue swabs were collected before sputum sample. The two swab heads were then pooled in Tris-EDTA-Tween (TET) buffer and processed for Ultra testing using a 2:1 sample reagent (SR)-to-sample ratio (466 µL SR + 234 µL sample). A total of 500 µL of the mixture was loaded into a cartridge prefilled with 1.5 mL of buffer, resulting in a final volume of 2 mL and a final SR concentration of 16.6%. This customized SR-based processing protocol was adopted to enhance diagnostic yield, informed by prior evidence of improved sensitivity in an analytical validation study (17).

Two early-morning sputum samples were collected on two consecutive days. The first sputum sample was tested immediately using Ultra. The second sample was kept at room temperature in a private collection area at the study sites for up to 2 hours before refrigeration. Subsequently, it was stored at –20°C in the prison laboratory for 4–8 weeks before being transported in a cold box to the Ethiopian Public Health Institute (EPHI) in Addis Ababa for culture. Culture was performed using the Mycobacterial Growth Indicator Tube (MGIT 960; Becton Dickinson, Franklin Lakes, NJ, USA) liquid culture system (18). Cultures were presumptively identified as *Mycobacterium tuberculosis* complex (MTBC) based on the presence of characteristic clumps and serpentine cords on Ziehl-Neelsen smears, and only those showing characteristic cording were subsequently confirmed as MTBC using the Capilia TB-Neo rapid antigen test (TAUNS, Japan) (19).

Demographic and clinical data were collected using standardized questionnaires. The diagnostic accuracy of tongue swab Ultra was evaluated by estimating sensitivity, specificity, positive predictive value, and negative predictive value, each with 95% confidence intervals (CIs). Data were entered into a REDCap database and analyzed using SPSS version 23 (20).

**Fig 1:**
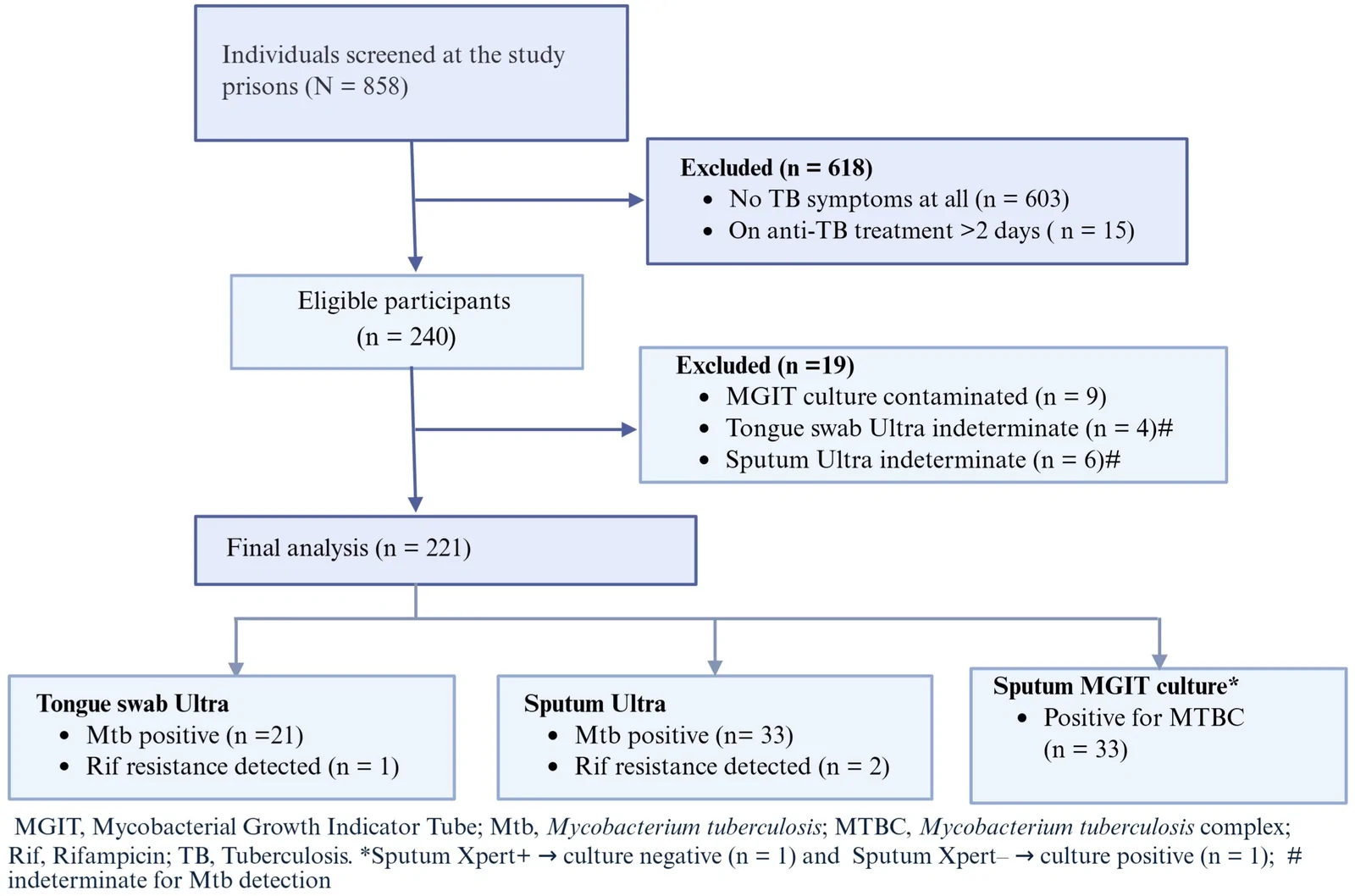
Participant enrollment and laboratory testing flow diagram for the evaluation of tongue swab Ultra for TB diagnosis in Ethiopian prisons, using sputum Ultra and culture as the composite reference standard.

A total of 858 incarcerated men were screened for TB symptoms, of whom 240 were eligible for the study, and a total of 221 were included in the final analysis. Among the 33 TB cases, there was concordance between sputum Ultra and liquid culture in 31 cases. Two cases showed discordant results: one was Ultra-positive/culture-negative, and the other was Ultra-negative/culture-positive (Fig. 1).

The majority (70.6%) of participants were recruited from Kality Federal Prison, and 63.3% were identified through our monthly active screening. The mean age was 30.5 years (SD ±9.4), and the median body mass index was 22.1 kg/m² (IQR 20.4–23.8); 9.0% were underweight. HIV prevalence was low (3.6%), and 2.7% reported a previous history of TB (Table 1).

**TABLE 1:** Demographic and clinical characteristics of participants for the evaluation of tongue swab Ultra for TB diagnosis in Ethiopian prisons, using sputum Ultra and culture as the composite reference standard^*a*^

Variable	Total *N* = 221	TB negative *n* = 187	MRS (sputum Ultra and/or culture positive) *n* = 34
Prison site: *n* (%)			
Kality Prison	162 (73.3)	140 (86.4)	22 (13.6)
Kilinto Detention Center	30 (13.6)	22 (73.3)	8 (26.7)
Ziway Prison	29 (13.1)	25 (86.2)	4 (13.8)
Age (years), mean ± SD	30.5 (9.4)	30.7 (9.5)	29.1 (8.5)
Enrollment strategy: *n* (%)			
Active screening	140 (63.3)	124 (88.6)	16 (11.4)
Contact tracing	33 (15.0)	27 (81.8)	6 (18.2)
Self-initiated care seeking	48 (21.7)	36 (75.0)	12 (25.0)
Educational status: *n* (%)			
Unable to read and write	20 (9.0)	13 (65.0)	7 (35.0)
Primary	97 (43.9)	79 (81.4)	18 (18.6)
Secondary	80 (36.2)	72 (90.0)	8 (10.0)
Higher education (college/university)	24 (10.9)	23 (95.8)	1 (4.2)
BMI, median (IQR)	22.1 (20.4-23.8)	22.4 **(**20.8-24.0**)**	19.9 (18.2-21.7)
Underweight (BMI <18.5)	20 (9.0)	8 (40.0)	12 (60.0)
Duration of incarceration (years) – Median (IQR)	2.1 (0-3.4)	2.4 (1.2-3.7)	1.1 (0-2.3)
Duration of incarceration: *n* (%)			
**<**6 months	7 (3.2)	7 (100.0)	0 (0.0)
6–12 months	41 (18.6)	25 (61.0)	16 (39.0)
≥12 months	173 (78.2)	155 (89.6)	18 (10.4)
History of TB: *n* (%)			
Yes	6 (2.7)	2 (33.3)	4 (66.7)
No	215 (97.3)	185 (86.0)	30 (14.0)
Shared a cell with TB patient: *n* (%)			
Yes	53 (24.0)	46 (86.8)	7 (13.2)
No	151 (68.3)	133 (60.2)	18 (39.8)
Do not know	17 (7.7)	8 (47.0)	9 (53.0)
TB symptoms: *n* (%)			
Cough ≥2 weeks	89 (40.3)	61 (68.5)	28 (31.5)
Cough <2 weeks	132 (59.7)	126 (95.4)	6 (4.6)
Fever of any duration	84 (38.0)	58 (69.0)	26 (31.0)
Night sweats of any duration	72 (32.6)	40 (55.6)	32 (44.4)
Weight loss	54 (24.4)	29 (53.7)	25 (46.3)
Hemoptysis	6 (2.7)	1 (16.7)	5 (83.3)
HIV status: *n* (%)			
With HIV	8 (3.6)	4 (50.0)	4 (50.0)
Without HIV	107 (48.4)	9 (8.4)	98 (91.6)
Unknown/Not tested	106 (48.0)	87 (82.0)	20 (18.9)

^*a*^ BMI, body mass index; HIV, human immunodeficiency virus; IQR, interquartile range; MGIT, mycobacterial growth indicator tube; MRS, microbiological reference standard; SD, standard deviation.

Relative to sputum Ultra, tongue swab Ultra demonstrated a sensitivity of 63.6% (21/33; 95% CI: 45.1%–79.6%) and a specificity of 100%. Against the MRS, the sensitivity was 61.8% (21/34; 95% CI: 44.7%–76.6%), with specificity remaining 100% (Table 2).

**TABLE 2:** Diagnostic accuracy of tongue swab Ultra for TB diagnosis in Ethiopian prisons compared with sputum Ultra and culture as the composite reference standard^*a*^

Parameter	Sputum Xpert Ultra reference standard *n*/*N*, % (95% CI)	Microbiologic reference standard *n*/*N*, % (95% CI)
Sensitivity	21/33, 63.6 (45.1–79.6)	21/34, 61.8 (44.7–76.6)
Specificity	188/188, 100 (98.1–100)	187/187, 100 (97.9–100)
PPV	21/21, 100 (83.9–100)	21/21, 100 (83.9–100)
NPV	188/200, 94 (89.7–96.8)	187/200, 93.5 (89.1–96.3)

^*a*^ CI, confidence interval; PPV, positive predictive value; NPV, negative predictive value.

Sensitivity varied according to sputum Ultra bacillary load: 100.0% (9/9) for medium or high loads, 81.8% (9/11) for low loads, 50.0% (3/6) for very low loads, and 0.0% (0/7) for “trace” loads.

Overall, tongue swab testing with Ultra using a customized SR-based processing method showed modest sensitivity but excellent specificity for TB diagnosis among incarcerated men in Ethiopian prisons. All TB cases with medium or high sputum bacillary loads were detected by tongue swab Ultra, indicating a strong influence of bacillary burden on test performance. Tongue swab Ultra also detected 70.6% (12/17) of cases with low or very low bacillary loads, suggesting potential utility even in paucibacillary disease. However, tongue swab Ultra detected none of the seven cases with a “trace” sputum bacillary load. The overall sensitivity indicates that tongue swab testing alone is not sufficient as a stand-alone test for TB diagnosis.

These findings have important implications for TB control in Ethiopian prisons and similar high-burden settings in sub-Saharan Africa, where diagnostic access is often constrained by limited infrastructure, suboptimal infection control practices, and the practical difficulties of sputum collection (4, 6). Tongue swabs help improve access to TB diagnosis and increase TB case detection in prisons. Although sensitivity was modest, broader uptake of screening and improved access to testing may enhance overall case detection, particularly among individuals unable or unwilling to produce sputum (21).

Operationally, tongue swab collection was feasible in this study, with all eligible participants successfully providing samples. Tongue swabs have previously been shown to be well accepted among study participants and require minimal infection control infrastructure, suggesting their potential for high uptake in prisons (22).

Limitations include exclusion of individuals with asymptomatic (subclinical) TB and incomplete HIV data due to relianceon self-report. Sputum specimens were frozen before culture processing, which may have reduced Mtb viability and potentially decreased culture sensitivity, particularly among samples with low bacillary loads. Subgroup analyses by bacillary load were limited by small sample sizes. Finally, alternative tongue swab processing methods were not evaluated, limiting attribution of performance to our customized protocol.

In conclusion, tongue swab testing using Ultra is a feasible adjunct diagnostic tool for TB detection in prisons, with high specificity and moderate sensitivity. Larger studies are needed to assess its performance in subclinical TB cases, optimize sensitivity for “trace” bacillary loads, and evaluate cost-effectiveness in this population.

## Supplementary Material

Reviewer comments

## Data Availability

The data sets generated and/or analyzed during the current study are not publicly available but are available from the corresponding author on reasonable request.

## SUPPLEMENTAL MATERIAL

Supplemental Material spectrum.00840-26-s0001.docx Supplemental Materials and Methods.

OPEN PEER REVIEW reviewer-comments.pdf An accounting of the reviewer comments and feedback.

ASM does not own the copyrights to Supplemental Material that may be linked to, or accessed through, an article. The authors have granted ASM a non-exclusive, world-wide license to publish the Supplemental Material files. Please contact the corresponding author directly for reuse.
