# Supplementary material for: Tongue swab Xpert MTB/RIF Ultra for tuberculosis diagnosis in Ethiopian prisons
Source: Microbiol Spectr. 2026 Aug 17;14(10):e00840-26. doi: 10.1128/spectrum.00840-26 (PMC13688924; doi:10.1128/spectrum.00840-26)
Supplement: Supplementary file 1 [file reviewer-comments.pdf]

ASM | Microbiology Spectrum

Oral Swab Xpert MTB/RIF Ultra for Tuberculosis Diagnosis in Ethiopian Prisons

Kelemework Adane, Russell Kempker, Kidist Bobosha, Abyot Meaza, and Henry Blumberg

Corresponding Author(s): Kelemework Adane, Addis Ababa University School of Medicine

Review Timeline:

Submission Date:	March 10, 2026
Editorial Decision:	April 24, 2026
Revision Received:	June 23, 2026
Accepted:	July 8, 2026

Editor: Victoria Campodónico

Reviewer(s): Disclosure of reviewer identity is with reference to reviewer comments included in decision letter(s). The following individuals involved in review of your submission have agreed to reveal their identity: Harley T Harris (Reviewer #1); Melissa Richard-Greenblatt (Reviewer #2)

Transaction Report:

(Note: With the exception of the correction of typographical or spelling errors that could be a source of ambiguity, letters and reports are not edited. The original formatting of letters and referee reports may not be reflected in this compilation.)

DOI: https://doi.org/10.1128/spectrum.00840-26

Re: Spectrum00840-26 (Oral Swab Xpert MTB/RIF Ultra for Tuberculosis Diagnosis in Ethiopian Prisons)

Dear Dr. Kelemework Adane:

Thank you for the privilege of reviewing your work. Below you will find my comments, instructions from the Spectrum editorial office, and the reviewer comments.

In addition to responding to the reviewers comments, due to the limited findings of this manuscript, please modify the manuscript and resubmit it as an observation, not a research paper.

Please return the manuscript within 60 days; if you cannot complete the modification within this time period, please contact me. If you do not wish to modify the manuscript and prefer to submit it to another journal, notify me immediately so that the manuscript may be formally withdrawn from consideration by Spectrum.

Revision Guidelines
To submit your modified manuscript, log into the submission site at https://spectrum.msubmit.net/cgi-bin/main.plex. Go to Author Tasks and click the appropriate manuscript title to begin. The information you entered when you first submitted the paper will be displayed; update this as necessary. Note the following requirements:

• Upload point-by-point responses to the issues raised by the reviewers in a file named "Response to Reviewers," NOT in your cover letter.
• Upload a compare copy of the manuscript (without figures) as a "Marked-Up Manuscript" file.
• Upload a clean .DOC/.DOCX version of the revised manuscript and remove the previous version.
• Each figure must be uploaded as a separate, editable, high-resolution file (TIFF or EPS preferred), and any multipanel figures must be assembled into one file.
• Any supplemental material intended for posting by ASM should be uploaded with their legends separate from the main manuscript. You can combine all supplemental material into one file (preferred) or split it into a maximum of 10 files with all associated legends included.

For complete guidelines on revision requirements, see our Submission and Review Process webpage. Submission of a paper that does not conform to guidelines may delay acceptance of your manuscript.

Data availability: ASM policy requires that data be available to the public upon online posting of the article, so please verify all links to sequence records, if present, and make sure that each number retrieves the full record of the data. If a new accession number is not linked or a link is broken, provide Spectrum production staff with the correct URL for the record. If the accession numbers for new data are not publicly accessible before the expected online posting of the article, publication may be delayed; please contact production staff (Spectrum@asmusa.org) immediately with the expected release date.

Publication Fees: For information on publication fees and which article types are subject to charges, visit our website. If your manuscript is accepted for publication and any fees apply, you will be contacted separately about payment during the production process; please follow the instructions in that e-mail. Arrangements for payment must be made before your article is published.

ASM Membership: Corresponding authors may join or renew ASM membership to obtain discounts on publication fees. Need to upgrade your membership level? Please contact Customer Service at Service@asmusa.org.

The ASM Journals program strives for constant improvement in our submission and publication process. Please tell us how we can improve your experience by taking this quick Author Survey.

Thank you for submitting your paper to Spectrum.

Sincerely,
Victoria Campodónico
Editor
Microbiology Spectrum

Reviewer #1 (Comments for the Author):

Thank you to the authors for sharing this highly applicable work. Below are some minor comments that need attention:
• There is some discrepancy in the sample reagent (SR) to sample ratio throughout the paper that needs to be addressed to properly reflect the methodology. In the abstract (line 62) this is described as 2:1 sample to RS ratio which does not match to the methods section (line 212-213). Also in the methods, please clarify in line 214 the final SR concentration. Per the previous

line of "466 uL SR + 234 TET" is a 2:1 SR: sample ratio and is 66%. I believe 16.6% is reflecting the final concentration after the 500 uL into 1.5 mL dilution in the cartridge. If this is correct, recommend moving final concentration percentage to end of paragraph to avoid confusion. Also, for clarity the ratio should be presented in the same order throughout the paper i.e. all SR: sample.

• Line 88: missing percentage symbol for prevalence "3.5%"
• Line 174-175: Here authors say no entry screening was done [by OSA?] which seems to conflict with line 142-143 which indicate entry screening is a key part of the study. Please clarify or adjust text to best reflect true practice
• Line 191: This is a bit confusing to read. Please clarify if 2 consecutive sputum samples were collected the same morning or if morning sputum samples were collected on two consecutive days.
• Line 260 and Table 1: the data here does not match the table. Adjust accordingly to correct data
• Line 261 and Table 1: given the explanation in the methods section it is not necessary to restate all study participants were male.

Reviewer #2 (Comments for the Author):

General
The following study assesses oral swabs as an alternative for TB evaluations in Ethiopian prisons. The findings from the study capture an important population impacted by TB that is greatly underserved. The manuscript can assist in addressing some of the needs of prison populations.

The authors need to identify a story they are interested in telling the reader with the data they have. It was very challenging to follow the rationale of the study and appreciate the implications of the use oral swabs in Ethiopian prisons. Many readers will not appreciate the nuances and challenges with diagnostics access in these settings. It would be great to provide explicit details on how oral swabs will improve TB evaluations for this population.

Major comments:
- I would frame the positioning of oral swabs in your manuscript as a diagnostic since you are using a testing method and collection approach with high specificity and lower sensitivity.

Abstract
- Introduction to paragraph discusses need for sputum-free diagnostic approaches for subclinical TB, yet the participants in the study were symptomatic with cough which is not considered subclinical TB
- Start of abstract could rather discuss need for non-sputum-based TB screening tools in prisons for specific populations and logistical reasons (infection control related to aerosolization of expectoration)
- State a two swab collection - with the details of collection (early morning followed by an additional swab collected 30 minutes later)
- Line 61: state that these are specifically incarcerated men and maintain this throughout the manuscript
- Line 73: 61-63% sensitivity is not considered moderate-to-high sensitivity. Rather, oral swabs can improve access for TB diagnosis.

Introduction
The introduction requires attention to adequately lead up to the rationale of the study and provide the necessary background. Here are some suggestions below:
- Line 97: the way the sentence is currently written, make it sound like we are missing most TB cases due to patients not being able to expectorate. This may be true for certain patient populations (children and PLWHIV); however, the diagnostic gap is largely due to inaccessibility to diagnostics generally.
- I would focus your introduction on how oral swabs can improve access to diagnostics in prisons. For example, PLWHIV often unable to expectorate as well as infection control (related to not having designated spaces for individuals expectorating)
- It might be worth mentioning HIV rates in Ethiopian prisons and how tongue swabs could potentially support diagnosis in these individuals and other information on infection control measures and safety surrounding the collection of sputum samples
- Since you are not evaluating individuals with subclinical disease, I would leave this out of the introduction
- Line 100: Sentence is saying two different things here - first it sounds like you are mentioning infection control needs for sputum collection in prisons, which is a great point and you should mention from a specimen collection perspective oral swabs are safer. The second part of the sentence (line 101) about segregating symptomatic individuals, it is unclear how this is relevant to your study. I would remove.
- Line 107-108: would state OSA with Xpert Ultra has not been systematically evaluated for TB diagnosis in prison settings.
- Line 118: please include references for customized oral swab processing approaches
- Line 117-126: Your study is not evaluating approaches in oral swab processing. Please remove the discussion of customized approaches from your introduction. This could be touched upon as a way to improve sensitivity in the discussion but it is not warranted here.
- The lead up to the rationale of your study is not clear and the way it is currently written sounds like you are evaluating different oral swab processing approaches
Methods

- Line 182: Please define a spot swab, or remove "spot" and state an additional swab 30 minutes later
- Line 185: Please clarify if swabs were placed into separate collection containers.
- Line 186: please specify the concentrations of each component and pH of the TET buffer
- Line 192: Freezing samples decreases viability of mycobacteria - in fact, we reject specimens in my mycobacteriology reference laboratory that have previously been frozen. How and for how long were the specimens at room temperature and/or refrigerated before being placed at -20C?
- Line 192: There can certainly be differences in bacterial loads between specimens and using two different specimens can lead to discrepant results. Performing Ultra testing on all first morning sputum samples may have biased results and should have been randomized.
- Line 197-198: Please describe what approach was used for Xpert - raw sputum or sedimented. If sedimented, please indicate what volume and if the volume was standardized across participants?
- Line 207: Please clarify how long were BHI plates incubated for. Generally, ZN is used to look for contamination of non-mycobacteria. Please clarify if ZN was also used to look for contamination with non-mycobacteria.
- Line 207: Please clarify if only those cultures positive for cording were sent for further identification
- Line 208: please indicate the manufacturer for Capilia TB-Neo rapid test
- Line 212: Stating swab type implies these are different types of swabs rather than to collections periods. Please change wording.
- Line 215: rather than stating the LoD details, can just mention that this protocol has been found to have greater sensitivity (reference) and therefore was used to improve diagnostic yield in the study.
- Line 218: A different buffer was used (TET vs SR) and volume (unknown for sputum vs 0.5 mL) for oral and sputum samples - this generates challenges for comparing performance due to additional differences in specimen processing
- Line 219: Was the oral testing performed with Xpert SR or TET. Lines 212 and 220 are challenging to understand and seem to contradict one another.

Results
- Please provide the performance of sputum Ultra compared to sputum culture
- Line 242: Please restate why only 221 participants were included in the final analysis
- Line 249: I don't think we need to include the finding of the NTM isolate
- Figure 1 title needs to be improved and should be able to stand alone from the text
- Table 1 title should be able to stand alone from the text
- Line 277: Please indicate that oral swab Ultra sensitivity was improved
- Line 283: I appreciate that you are referring to ultra bacillary loads, but please make it clear here and throughout the text that this is related to Ultra semi-quantitation to avoid confusion from culture or AFB smear. You could help clarify upfront by your subtitle for the section as "Diagnostic Accuracy by Ultra Semi-quantitative grade"
- Line 287: not sure how TB-related symptoms fits into this section. Please remove the last sentence of this paragraph and also consider removing from the table. The way the paper is currently written, I am not sure of the relevance of including the # symptoms and Ct values. If you leave this in, it should be built more into your paper with background and rationale for it.
- Table 3: Missing a bracket with (4/4; remain consistent with number of decimal places used. The mean number of symptoms uses a decimal; however, % detected by oral swabs do not for Ultra grades.
- The relevance of the comparison in Ct values (Figure 3) between Ultra sputum and oral swabs is unclear considering different volumes of specimen were included and the authors did not describe if processed or unprocessed sputum was used
- Please briefly comment on

Discussion
- The discussion should be substantially shortened and reworked with more emphasis on implications of findings and benefits to oral swab use in prisons rather than comparisons with diagnostic yield of other studies
- Line 303: the first sentence should more accurately reflect the findings of the study, for example you could state "The following study demonstrated that oral swabs using Xpert MTB/RIF Ultra provides modest overall sensitivity but excellent specificity for pulmonary TB diagnosis among incarcerated men in high-burden Ethiopian prison settings"
- In the intro paragraph of your discussion, you can state the performance characteristics but requires emphasis that oral swabs are not sufficient as stand alone tests for TB case detection
- Limitations of the study should also include: absence of subclinical TB, which would assist in understand the role of oral swabs in screening strategies; HIV status was self-reported and incomplete, limiting assessment of performance in PLWHIV, who often present with lower bacillary burden and are unable to expectorate; subgroup analyses by bacillary load were small numbers, which can result in imprecise estimates. Samples were frozen prior to setting up culture which could have reduced sensitivity of culture, especially in specimens with lower bacterial loads and resulted in artificially elevated sensitivity findings of oral swabs
- Please mention how the study did not evaluate cost-effectiveness, patient acceptability, or impact on transmission which are important considerations for implementation in Ethiopian prisons
- Line 394-395: Please reference studies that have demonstrated no different in oral swab sensitivity between sexes. If none exists, please remove statement that there are unlikely to be sex differences.

Journal Name: Microbiology Spectrum

Manuscript Number: Spectrum00840-26

Manuscript Title: Oral Swab Xpert MTB/RIF Ultra for Tuberculosis Diagnosis in Ethiopian Prisons

Overall Impressions and comments to editor:

This paper presents a well-designed study to assess the clinical performance of oral swab testing for *M. tuberculosis* by Xpert Ultra assay. While other similar studies have been previously done, this study combines being in a higher prevalence setting (prison in sub-Saharan Africa) with modified swab processing procedure. Overall, the paper is very strong and will be a good addition to the field. There are some minor edits that need to be made to correct some errors and improve clarity.

Comments for Authors:

Thank you to the authors for sharing this highly applicable work. Below are some minor comments that need attention:

- There is some discrepancy in the sample reagent (SR) to sample ratio throughout the paper that needs to be addressed to properly reflect the methodology. In the abstract (line 62) this is described as 2:1 sample to RS ratio which does not match to the methods section (line 212-213). Also in the methods, please clarify in line 214 the final SR concentration. Per the previous line of "466 uL SR + 234 TET" is a 2:1 SR: sample ratio and is 66%. I believe 16.6% is reflecting the final concentration after the 500 uL into 1.5 mL dilution in the cartridge. If this is correct, recommend moving final concentration percentage to end of paragraph to avoid confusion. Also, for clarity the ratio should be presented in the same order throughout the paper i.e. all SR: sample.
- Line 88: missing percentage symbol for prevalence "3.5%"
- Line 174-175: Here authors say no entry screening was done [by OSA?] which seems to conflict with line 142-143 which indicate entry screening is a key part of the study. Please clarify or adjust text to best reflect true practice
- Line 191: This is a bit confusing to read. Please clarify if 2 consecutive sputum samples were collected the same morning or if morning sputum samples were collected on two consecutive days.
- Line 260 and Table 1: the data here does not match the table. Adjust accordingly to correct data
- Line 261 and Table 1: given the explanation in the methods section it is not necessary to restate all study participants were male.

June 20, 2026

Response to Reviewer Comments

I appreciate the reviewers' constructive comments on my submitted manuscript (Spectrum00840-26). Below, I provide responses outlining the revisions made to address each comment.

Reviewer #1 (Comments for the Author):

Thank you to the authors for sharing this highly applicable work. Below are some minor comments that need attention:

1. There is some discrepancy in the sample reagent (SR) to sample ratio throughout the paper that needs to be addressed to properly reflect the methodology. In the abstract (line 62) this is described as 2:1 sample to RS ratio which does not match to the methods section (line 212-213). Also in the methods, please clarify in line 214 the final SR concentration. Per the previous line of "466 uL SR + 234 TET" is a 2:1 SR: sample ratio and is 66%. I believe 16.6% is reflecting the final concentration after the 500 uL into 1.5 mL dilution in the cartridge. If this is correct, recommend moving final concentration percentage to end of paragraph to avoid confusion. Also, for clarity the ratio should be presented in the same order throughout the paper i.e. all SR: sample.

   Revision: **Modifications made at each section in the manuscript.** Please see lines 41-42, 266-267.
2. Line 88: missing percentage symbol for prevalence "3.5%"

   Revision**: Now fixed.** Please see line 93.
3. Line 174-175: Here authors say no entry screening was done [by OSA?] which seems to conflict with line 142-143 which indicate entry screening is a key part of the study. Please clarify or adjust text to best reflect true practice.

   Revision: **The description is now adjusted.** Please see lines 176-177.
4. Line 191: This is a bit confusing to read. Please clarify if 2 consecutive sputum samples were collected the same morning or if morning sputum samples were collected on two consecutive days.

   Revision: **This is now corrected**. Please see lines 127- 228.
5. Line 260 and Table 1: the data here does not match the table. Adjust accordingly to correct data

   Revision**: This is now corrected.** Please see table 1.
6. Line 261 and Table 1: given the explanation in the methods section it is not necessary to restate all study participants were male

   Revision: **This is now corrected.** Please see table 1 and also line 318.

Reviewer #2 (Comments for the Author):

General

The following study assesses oral swabs as an alternative for TB evaluations in Ethiopian prisons. The findings from the study capture an important population impacted by TB that is greatly underserved. The manuscript can assist in addressing some of the needs of prison populations.

The authors need to identify a story they are interested in telling the reader with the data they have. It was very challenging to follow the rationale of the study and appreciate the implications of the use oral swabs in Ethiopian prisons. Many readers will not appreciate the nuances and challenges with diagnostics access in these settings. It would be great to provide explicit details on how oral swabs will improve TB evaluations for this population.

Revision: **The introduction has been revised based on these comments.** <u>Please see the revised introduction.</u>

<u>Major comments:</u> I would frame the positioning of oral swabs in your manuscript as a diagnostic since you are using a testing method and collection approach with high specificity and lower sensitivity**.**

Revision: **Revisions have been made to the introduction and other sections accordingly.** <u>Please see the revised introduction, abstract, and other sections.</u>

Abstract.

- Introduction to paragraph discusses need for sputum-free diagnostic approaches for subclinical TB, yet the participants in the study were symptomatic with cough which is not considered subclinical TB. Start of abstract could rather discuss need for non-sputum-based TB screening tools in prisons for specific populations and logistical reasons (infection control related to aerosolization of expectoration)

  Revision: **This has been addressed. The description of subclinical TB has been removed, and the abstract has been revised and restructured in line with the comments.**

- State a two-swab collection - with the details of collection (early morning followed by an additional swab collected 30 minutes later)

  Revision: **This has been addressed.** <u>Please see line 216.</u>

- Line 61: state that these are specifically incarcerated men and maintain this throughout the manuscript

  Revision: **This has been addressed.** <u>Please see lines 39, 59, etc.</u>

- Line 73: 61-63% sensitivity is not considered moderate-to-high sensitivity. Rather, oral swabs can improve access for TB diagnosis.

Revision: **This has been addressed** <u>(lines 56–58 and elsewhere in the manuscript</u>**). The importance of improving access to TB diagnosis has now been emphasized throughout the manuscript.**

Introduction

The introduction requires attention to adequately lead up to the rationale of the study and provide the necessary background. Here are some suggestions below:

- Line 97: the way the sentence is currently written, make it sound like we are missing most TB cases due to patients not being able to expectorate. This may be true for certain patient populations (children and PLWHIV); however, the diagnostic gap is largely due to inaccessibility to diagnostics generally.

  Revision: **The introduction has been revised, and these points have been duly considered.**
- I would focus your introduction on how oral swabs can improve access to diagnostics in prisons. For example, PLWHIV often unable to expectorate as well as infection control (related to not having designated spaces for individuals expectorating)

  Revision: **The introduction has been revised, and these points have been duly considered.**
- It might be worth mentioning HIV rates in Ethiopian prisons and how tongue swabs could potentially support diagnosis in these individuals and other information on infection control measures and safety surrounding the collection of sputum samples

  Revision: **The introduction has been revised, and these points have been duly considered.**
- Since you are not evaluating individuals with subclinical disease, I would leave this out of the introduction

  Revision**: This has now been removed from the introduction, and the focus has been shifted to the suggested points, which are very helpful and greatly appreciated**.
- Line 100: Sentence is saying two different things here - first it sounds like you are mentioning infection control needs for sputum collection in prisons, which is a great point and you should mention from a specimen collection perspective oral swabs are safer. The second part of the sentence (line 101) about segregating symptomatic individuals, it is unclear how this is relevant to your study. I would remove.

  Revision: **The introduction has been revised, and these points have been duly considered.**
- Line 107-108: would state OSA with Xpert Ultra has not been systematically evaluated for TB diagnosis in prison settings.

  Revision: **I have now added this description to the appropriate section of the introduction.** Please see lines 124-125.
- Line 118: please include references for customized oral swab processing approaches

Revision: **This section has been modified, and the focus on customized oral swab processing has been de-emphasized. However, the relevant reference has been cited in the Methods section where such protocols are described.** Please see line 269**.**

- Line 117-126: Your study is not evaluating approaches in oral swab processing. Please remove the discussion of customized approaches from your introduction. This could be touched upon as a way to improve sensitivity in the discussion but it is not warranted here

Revision: **This has now been revised as suggested, and the discussion of customized approaches has been removed.**

- The lead up to the rationale of your study is not clear and the way it is currently written sounds like you are evaluating different oral swab processing approaches

  Revision: **This has now been revised as suggested.**

Methods

- Line 182: Please define a spot swab, or remove "spot" and state an additional swab 30 minutes later

  Revision: **This has now been corrected.** Please see lines 215-216.
- Line 185: Please clarify if swabs were placed into separate collection containers.

  Revision: **Both swab heads were placed in a single Falcon tube.** Please see lines 218-219.
- Line 186: please specify the concentrations of each component and pH of the TET buffer

  Revision: **The concentrations are now specified as suggested.** Please see lines 220-222.
- Line 192: Freezing samples decreases viability of mycobacteria - in fact, we reject specimens in my mycobacteriology reference laboratory that have previously been frozen. How and for how long were the specimens at room temperature and/or refrigerated before being placed at -20C?

  Revision: **The first sputum sample was tested immediately using Xpert Ultra. The second sample was kept at room temperature in a private collection area at the study sites for a maximum of two hours before refrigeration. Subsequently, it was stored at –20 °C in the prison laboratory for 4–8 weeks before being transported in a cold box to the Ethiopian Public Health Institute (EPHI) in Addis Ababa for culture processing. Please see lines 233-236.**

  Freezing may reduce mycobacterial viability; however, repeated thawing and refreezing was avoided. Freezing was unavoidable due to logistical constraints and the limited processing capacity at the reference laboratory at EPHI, which necessitated batch processing and required storage times before culture could be performed. In our study, only one participant whose sputum sample was Xpert Ultra–positive was culture-negative, while another participant who was Xpert Ultra–negative became culture-positive. This pattern suggests that, overall, our findings were unlikely to have been substantially affected by the freezing process.
- Line 192: There can certainly be differences in bacterial loads between specimens and using two different specimens can lead to discrepant results. Performing Ultra testing on all first morning sputum samples may have biased results and should have been randomized.

  Response: **We agree the possible differences in bacterial load between sputum specimens. However, in our study, both specimens were first-morning sputum samples collected on consecutive days rather than a spot specimen and a morning specimen, which may have reduced variability in bacillary load between specimens. We did not randomize specimen**

allocation because the study protocol required Xpert Ultra testing to be performed immediately on the first morning sputum sample after collection, while the second morning sample collected on the following day was reserved for culture. Delaying Ultra testing until collection of the second specimen was not feasible within the study workflow.

- Line 197-198: Please describe what approach was used for Xpert - raw sputum or sedimented. If sedimented, please indicate what volume and if the volume was standardized across participants?

  ***Revision:* Raw sputum was used for testing, and this has now clearly stated.** Please see line 239.

- Line 207: Please clarify how long were BHI plates incubated for. Generally, ZN is used to look for contamination of non-mycobacteria. Please clarify if ZN was also used to look for contamination with non-mycobacteria.

  ***Revision*: Yes, ZN staining was used for the presumptive identification of MTBC and to distinguish non-mycobacteria from potential contaminants. This has now been clearly described**.

- Line 207: Please clarify if only those cultures positive for cording were sent for further identification

  ***Revision:* Yes, only cultures showing characteristic cording on ZN smears were subjected to further identification using the Capilia TB-Neo rapid test. This has now been described.** Please see lines 252-260.

- Line 208: please indicate the manufacturer for Capilia TB-Neo rapid test

  ***Revision*: This has now been included.** Please see line 256**.**

- Line 212: Stating swab type implies these are different types of swabs rather than to collections periods. Please change wording.

  ***Revision*: This has now been corrected.** Please see lines 217-219.

- Line 215: rather than stating the LoD details, can just mention that this protocol has been found to have greater sensitivity (reference) and therefore was used to improve diagnostic yield in the study.

  ***Revision:* This has now been corrected as suggested.** Please see lines 268-269.

- Line 218: A different buffer was used (TET vs SR) and volume (unknown for sputum vs 0.5 mL) for oral and sputum samples - this generates challenges for comparing performance due to additional differences in specimen processing.

  ***Response:* Using different protocols for sputum and oral swab specimens is supported by the literature. The SR is routinely used in Xpert Ultra for sputum processing, where it facilitates liquefaction and mycobacterial inactivation. For sputum, approximately 2 mL of specimen (now indicated in the manuscript) can be directly mixed with SR without volume constraints.** Please see line 240.

In contrast, oral swabs contain a very small amount of material, requiring an initial elution step using TET buffer to release and dilute the specimen prior to SR addition. This step also allows adjustment of the final SR volume, reducing the amount of SR so that the relatively few bacilli in oral swabs are not adversely affected by the higher SR concentration that would result if the same procedure as sputum were used.

- Line 219: Was the oral testing performed with Xpert SR or TET. Lines 212 and 220 are challenging to understand and seem to contradict one another.
  Revision: This **has now been corrected.** Please see lines 265-267.

Results

- Please provide the performance of sputum Ultra compared to sputum culture
  Revision: This **has now been corrected.** Please see lines 340-342.
- Line 242: Please restate why only 221 participants were included in the final analysis
  Response: **As described in Lines 294–299 and indicated in Figure 1, this was due to contamination of the MGIT culture and indeterminate MTB results on Ultra.**
- Line 249: I don't think we need to include the finding of the NTM isolate
  Revision: This **has now been corrected.** Please see lines 301-302and figure 1.
- Figure 1 title needs to be improved and should be able to stand alone from the text
  Revision: **This has now been revised.** Please see lines 312-314.
- Table 1 title should be able to stand alone from the text- Line 277: Please indicate that oral swab Ultra sensitivity was improved
  Revision: **This has now been revised.** Please see lines 328-330.
- Line 283: I appreciate that you are referring to ultra bacillary loads, but please make it clear here and throughout the text that this is related to Ultra semi quantitation to avoid confusion from culture or AFB smear. You could help clarify upfront by your subtitle for the section as "Diagnostic Accuracy by Ultra Semi quantitative grade
  Revision: **This has now been revised.** Please see lines 350 and 352.
- Line 287: not sure how TB-related symptoms fits into this section. Please remove the last sentence of this paragraph and also consider removing from the table. The way the paper is currently written, I am not sure of the relevance of including the # symptoms and Ct values. If you leave this in, it should be built more into your paper with background and rationale for it
  Revision: This has now been revised; I have removed those contents**.** Please see lines 356 - 357 and Table 3.
- Table 3: Missing a bracket with (4/4; remain consistent with number of decimal places used. The mean number of symptoms uses a decimal; however, % detected by oral swabs do not for Ultra grades.
  Revision: **This has now been revised.** Please see table 3.

- The relevance of the comparison in Ct values (Figure 3) between Ultra sputum and oral swabs is unclear considering different volumes of specimen were included and the authors did not describe if processed or unprocessed sputum was used. Please briefly comment on
  ***Revision:* I have removed the content on CT values, and I agree that it is important to focus on the other key findings**. Please see lines 364-368 and figure 2 (deleted).

Discussion

- The discussion should be substantially shortened and reworked with more emphasis on implications of findings and benefits to oral swab use in prisons rather than comparisons with diagnostic yield of other studies
  ***Revision:* The discussion has now been extensively revised as per the suggestions.**
- Line 303: the first sentence should more accurately reflect the findings of the study, for example you could state "The following study demonstrated that oral swabs using Xpert MTB/RIF Ultra provides modest overall sensitivity but excellent specificity for pulmonary TB diagnosis among incarcerated men in high-burden Ethiopian prison settings"
  ***Revision*: This comment has now been incorporated.** Please see lines 374-378**.**
- In the intro paragraph of your discussion, you can state the performance characteristics but requires emphasis that oral swabs are not sufficient as standalone tests for TB case detection
  ***Revision*: This comment has now been incorporated.** Please see lines 379-381.
- Limitations of the study should also include: absence of subclinical TB, which would assist in understand the role of oral swabs in screening strategies; HIV status was self-reported and incomplete, limiting assessment of performance in PLWHIV, who often present with lower bacillary burden and are unable to expectorate; subgroup analyses by bacillary load were small numbers, which can result in imprecise estimates. Samples were frozen prior to setting up culture which could have reduced sensitivity of culture, especially in specimens with lower bacterial loads and resulted in artificially elevated sensitivity findings of oral swabs
  ***Revision:* The limitations section has now been revised based on the suggestions provided.** Please see lines 441-452.
- Please mention how the study did not evaluate cost-effectiveness, patient acceptability, or impact on transmission which are important considerations for implementation in Ethiopian prisons
  ***Response/Revision:* We did not assess cost-effectiveness, as this was a pilot study focused on feasibility and diagnostic accuracy. This has now been acknowledged in the limitations section. Patient acceptability was also not systematically assessed, as the primary focus of the study was feasibility. However, all eligible participants successfully provided oral swab samples, and none refused the procedure, suggesting a high level of acceptability**

Line 394-395: Please reference studies that have demonstrated no different in oral swab sensitivity between sexes. If none exists, please remove statement that there are unlikely to be sex differences**.**

Revision: **There are no references showing such a difference; hence, I removed that part as suggested.** Please see line 436.

Re: Spectrum00840-26R1 (Oral Swab Xpert MTB/RIF Ultra for Tuberculosis Diagnosis in Ethiopian Prisons)

Dear Dr. Kelemework Adane:

Your manuscript has been accepted, and I am forwarding it to the ASM production staff for publication. However, there are still minor edits needed. Please, make sure you make all the modifications suggested by the reviewers before resubmitting for publication. Your paper will first be checked to make sure all elements meet the technical requirements. ASM staff will contact you if anything needs to be revised before copyediting and production can begin. Otherwise, you will be notified when your proofs are ready to be viewed.

Data Availability: ASM policy requires that data be available to the public upon online posting of the article, so please verify all links to sequence records, if present, and make sure that each number retrieves the full record of the data. If a new accession number is not linked or a link is broken, provide production staff with the correct URL for the record. If the accession numbers for new data are not publicly accessible before the expected online posting of the article, publication may be delayed; please contact ASM production staff immediately with the expected release date.

Publication Fees: For information on publication fees and which article types have charges, please visit our website. We have partnered with Copyright Clearance Center (CCC) to collect author charges. If fees apply to your paper, you will receive a message from no-reply@copyright.com with further instructions. For questions related to paying charges through RightsLink, please contact CCC at ASM_Support@copyright.com or toll free at +1-877-622-5543. CCC makes every attempt to respond to all emails within 24 hours.

ASM Membership: Corresponding authors may join or renew ASM membership to obtain discounts on publication fees. Need to upgrade your membership level? Please contact Customer Service at Service@asmusa.org.

Embargo Policy: A press release may be issued as soon as the manuscript is posted on the Spectrum Latest Articles webpage. The corresponding author will receive an email with the subject line "ASM Journals Author Services Notification" when the article is available online.

The ASM Journals program strives for constant improvement in our submission and publication process. Please tell us how we can improve your experience by taking this quick Author Survey.

Thank you for submitting your paper to Spectrum.

Sincerely,
Victoria Campodónico
Editor
Microbiology Spectrum

Reviewer #2 (Comments for the Author):

Thank you to the authors for incorporating the large number of suggestions in the last round of revisions. The manuscript is greatly improved and very reads well. I really enjoyed reading the revised manuscript. I have a few additional suggestions to further strengthen the content.

General:
- Sorry for not mentioning this earlier. I recently returned from an advanced TB diagnostics course where there was considerable discussion on the terminology of oral swabs vs tongue swabs, etc. Based on discussions with experts in the field, it is felt that the description of an oral swab should be more specific in studies. Please update throughout the text that this is a tongue swab.

Abstract:
- Line 27: remove "the" from the oral swab testing using...

Methods
- Line 73-74: Please briefly describe what was included as part of the active symptom screening. Was it only cough? Please clarify.
- Please indicate if there were any exclusion criteria.
- Line 85: Please specify when the two early-morning sputum samples were collected in relation to the tongue swabs
- Line 86: Please indicate for how long the specimen was refrigerated before being stored at -20c as well as state if putting into

the refrigerator was immediate or if there was a period of time specimens were at room temperature.
- Line 86: the viability of bacteria is known to be diminished by freezing specimens this may have caused a reduction in sensitivity of cultures, particularly at lower bacterial loads. Please expand on this as a major limitation of the study and this may have caused inflated performance characteristics of the tongue swab against the MRS on line 131-132.

Discussion:
- Line 127-128: please update the sentence to be something along the lines of: tongue swabs are non-invasive, have previously been identified as well accepted by study participants (the way it is currently phrased it sounds like it was something you measured in your study), and requires minimal infection control infrastructure, all of which may suggest high uptake of this collection method in prison settings.
