# Supplementary material for: Tongue swab Xpert MTB/RIF Ultra for tuberculosis diagnosis in Ethiopian prisons
Source: Microbiol Spectr. 2026 Aug 17;14(10):e00840-26. doi: 10.1128/spectrum.00840-26 (PMC13688924; doi:10.1128/spectrum.00840-26)
Supplement: Supplementary file 2 [file spectrum.00840-26-s0001.docx]

**MATERIALS AND METHODS**

**Study Design and Setting**

This was a cross-sectional study that prospectively enrolled participants from Kality Federal Prison and Kilinto Detention Center in Addis Ababa, as well as Ziway Prison, located approximately 170 km south of Addis Ababa. Kality Federal Prison houses approximately 4,500 incarcerated individuals, while Ziway Prison houses 2,742 individuals and accepts overflow transfers from Kality Federal Prison. Kilinto Detention Center is located near Kality Federal Prison and houses approximately 900 individuals. Healthcare services are provided by Kality Federal Prison Hospital and the Ziway Prison Health Center, each equipped with four-module GeneXpert instruments (Cepheid, Sunnyvale, CA, USA) for rapid TB diagnosis. Incarcerated individuals at Kilinto Detention Center utilize laboratory facilities at Kality Federal Prison Hospital. At the study sites, TB diagnosis relied primarily on passive case finding. All confirmed TB cases were isolated and received anti-TB treatment according to national guidelines.

**Study Population and Participant Enrollment**

**Study Population**

The study population consisted of adult incarcerated individuals from the selected prisons and the detention center. Eligible participants were adult incarcerated men (≥18 years) with cough of any duration, with or without other TB-related symptoms, who were willing and able to provide written informed consent and the required study specimens (sputum and tongue swab samples). Individuals who had received anti-TB treatment for more than two days before enrollment were excluded.

**Participant Enrollment**

Participant enrollment involved active case finding, contact investigation, and screening of individuals presenting to prison clinics with TB-related symptoms. Between January 15 and October 31, 2025, 858 incarcerated men were screened for eligibility from a combined prison population of 8,142 individuals across the three study sites. Most screening activities were conducted at Kality Federal Prison, where monthly active case-finding was implemented in selected prison cells. Two trained prison nurses conducted monthly cell-to-cell visits in randomly selected prison cells throughout the study period. During these visits, incarcerated individuals were screened for cough and other TB-related symptoms using a standardized screening protocol. Each month, a new set of cells was selected, and screening was conducted over two consecutive days. Incarcerated individuals identified as close contacts of confirmed TB cases were also screened throughout the study period. Close contacts were defined as incarcerated individuals who shared the same cell or living area with a confirmed TB case or had frequent and prolonged exposure (e.g., repeated daily contact within the same enclosed prison setting) before treatment initiation. Furthermore, individuals who visited the prison clinic with TB-related symptoms between monthly screening cycles were also assessed for study eligibility.

Screening at Ziway Prison and Kilinto Detention Center followed a similar approach; however, active screening was conducted only twice during the study period (May 5–7 and September 8–10, 2025). Contact tracing and assessment of incarcerated individuals who sought care passively were conducted continuously throughout the study period.

Following screening, incarcerated individuals meeting the inclusion criteria were enrolled in the study. Data were collected on socio-demographic and health characteristics, including age, sex, duration of imprisonment, history of sharing a cell with previously diagnosed TB patients, TB symptoms, past TB history, and HIV status. HIV status was obtained by self-report from participants; no HIV testing was performed as part of the study.

Screening was limited to resident incarcerated men living in the prisons. Entry screening of newly admitted individuals was not performed due to logistical constraints, and incarcerated women were not included. At Kality Federal Prison, women resided in a separate compound approximately 10 km from the men’s compound, and simultaneous screening of both compounds was not possible.

**Sample Collection**

Trained prison-based laboratory personnel at each study site collected the required specimens from all participants. Two tongue swabs were collected from each participant: the first early in the morning and the second 30 minutes later, before eating, drinking, or performing oral hygiene procedures. On each study day, tongue swabs were collected before sputum collection to minimize potential interference from sputum expectoration on oral specimen collection. Tongue swabs were collected by brushing the mid-dorsum of the tongue 10 times using FLOQSwabs (Tianzhu Sun Time Biotechnology, China). Both swab heads were placed in a single Falcon tube containing 234 µL of sterile 1× Tris-EDTA-Tween (TET) buffer and stored at 2–8 °C until processing within five days. The TET buffer had a final pH of 8.0 and consisted of 10 mM Tris-HCl (pH 8.0), 1 mM EDTA (pH 8.0), and 0.05% (v/v) Tween-20.

Previous studies have shown that processing two swabs together, rather than a single swab, can improve the sensitivity of TB detection (1). The 15–30-minute interval between swab collections allows replenishment of *Mycobacterium tuberculosis* (Mtb) on the tongue surface, ensuring independent and adequately loaded samples for testing (2).

Two early-morning sputum samples, each of sufficient volume (>3 mL), were collected on two consecutive days from each participant. The first sputum sample was tested immediately using Ultra. The second sample was kept at room temperature in a private collection area at the study sites for up to two hours before refrigeration. Subsequently, it was stored at –20 °C in the prison laboratory for 4–8 weeks before being transported in a cold box to the Ethiopian Public Health Institute (EPHI) in Addis Ababa for culture processing.

**Laboratory Analysis**

**Sputum Ultra**

Ultra testing was performed on the first morning raw sputum sample according to the manufacturer’s instructions. Briefly, approximately 2 mL of sputum was added to the Ultra Sample Reagent (SR) at a 2:1 ratio (SR to sample). After mixing and incubation, 2 mL of the treated mixture was loaded into the cartridge. The system automatically processes the sample within the cartridge and performs a hemi-nested qPCR targeting the *IS6110* and *IS1081* sequences, as well as the *rpoB* gene for rifampin resistance (3).

**Mycobacterial Growth Indicator Tube (MGIT) Culture**

MGIT 960 (Becton Dickinson, Franklin Lakes, NJ, USA) liquid culture was performed at the National Tuberculosis Research and Reference Laboratory, EPHI, on the second sputum sample according to the manufacturer’s instructions (4). Sputum samples were decontaminated using the NALC–NaOH method (0.25% N-acetyl-L-cysteine, 1% NaOH), and the resulting sediments were inoculated into MGIT 960 tubes. Cultures were presumptively identified as *Mycobacterium tuberculosis* complex (MTBC) based on acid-fast bacilli (AFB) morphology and characteristic serpentine cording on Ziehl–Neelsen staining. Cultures showing characteristic cording were subsequently confirmed as MTBC using the Capilia TB-Neo rapid antigen test (TAUNS, Japan) (5). Positive cultures were also inoculated onto Brain Heart Infusion (BHI) agar and incubated for 24 hours, and absence of growth was considered indicative of no detectable non-mycobacterial contamination. Laboratory personnel conducting culture testing were blinded to the tongue swab Ultra results.

**Tongue Swab Ultra**

Tongue swab Ultra testing was performed using a 2:1 ratio of SR to sample. Briefly, 466 µL of SR was added to 234 µL of TET buffer containing both swabs, giving a total volume of 700 µL. Then, 500 µL of the mixture was transferred into an Ultra cartridge prefilled with 1.5 mL of TET buffer (final volume, 2 mL), resulting in a final SR concentration of 16.6%; the mixture was then tested on Ultra at the prison laboratory. This protocol demonstrated greater sensitivity during analytical validation and was therefore used to improve the diagnostic yield of the study (6).

**Definitions**

A composite microbiological reference standard (MRS) consisting of sputum Ultra and MGIT culture was used. Microbiologically confirmed TB was defined as the detection of Mtb by MGIT culture and/or a positive sputum Ultra result at any semiquantitative level (high, medium, low, very low, or trace). All patients diagnosed with TB were promptly linked to appropriate treatment and care.

**Data Analysis**

A REDCap database was used for data entry, and SPSS version 23 was used for data analysis (7). The sensitivity and specificity of tongue swab Ultra were calculated using sputum Ultra and the MRS and are reported with 95% confidence intervals (CIs). Positive predictive value and negative predictive value were also estimated with corresponding 95% confidence intervals. In addition, the sensitivity of the tongue swab Ultra was calculated for those with sputum Ultra reference test across different bacillary loads.

**References**

1.	Andama A, Whitman GR, Crowder R, Reza TF, Jaganath D, Mulondo J, Nalugwa TK, Semitala FC, Worodria W, Cook C, Wood RC, Weigel KM, Olson AM, Lohmiller Shaw J, Kato-Maeda M, Denkinger CM, Nahid P, Cangelosi GA, Cattamanchi A. 2022. Accuracy of Tongue Swab Testing Using Xpert MTB-RIF Ultra for Tuberculosis Diagnosis. J Clin Microbiol 60:e0042122. https://doi.org/10.1128/jcm.00421-22

2.	Wood RC, Luabeya AK, Weigel KM, Wilbur AK, Jones-Engel L, Hatherill M, Cangelosi GA. 2015. Detection of Mycobacterium tuberculosis DNA on the oral mucosa of tuberculosis patients. Sci Rep 5: 8668. https://doi.org/10.1038/srep08668

3.	Chakravorty S, Simmons AM, Rowneki M, Parmar H, Cao Y, Ryan J, Banada PP, Deshpande S, Shenai S, Gall A, Glass J, Krieswirth B, Schumacher SG, Nabeta P, Tukvadze N, Rodrigues C, Skrahina A, Tagliani E, Cirillo DM, Davidow A, Denkinger CM, Persing D, Kwiatkowski R, Jones M, Alland D. 2017. The new Xpert MTB/RIF Ultra: improving detection of Mycobacterium tuberculosis and resistance to rifampin in an assay suitable for point-of-care testing. MBio 29:e00812-17. https://doi.org/10.1128/mbio.00812-1

4.	Siddiqi SH, Rüsch-Gerdes SJ. 2006. Mycobacteria Growth Indicator Tube (MGIT) culture and drug susceptibility demonstration project. Foundation for Innovative New Diagnostics (FIND), Geneva, Switzerland.

5.	Muyoyeta M, Mwanza WC, Kasese N, Cheeba-Lengwe M, Moyo M, Kaluba-Milimo D, Ayles H. 2013. Sensitivity, specificity, and reproducibility of the Capilia TB-Neo assay. J Clin Microbiol 51: 4237-9. https://doi.org/10.1128/jcm.02441-1

6.	Chilambi GS, Reiss R, Daivaa N, Banada P, De Vos M, Penn-Nicholson A, Alland D. 2025. Optimal processing of tongue swab samples for Mycobacterium tuberculosis detection by the Xpert MTB/RIF Ultra assay. Microbiol Spectr 13: e0240324.  https://doi.org/10.1128/spectrum.02403-24

7.	H Harris PA, Taylor R, Minor BL, Elliott V, Fernandez M, O'Neal L, McLeod L, Delacqua G, Delacqua F, Kirby J, Duda SN. 2019. The REDCap consortium: building an international community of software platform partners. J Biomed Inform 95: 103208. https://doi.org/10.1016/j.jbi.2019.103208
